# Leveraging transformers for semi-supervised pathogenicity prediction with soft labels

**DOI:** 10.1515/jib-2024-0047

**Published:** 2025-06-23

**Authors:** Pablo Enrique Guillem, Marco Zurdo-Tabernero, Noelia Egido Iglesias, Ángel Canal-Alonso, Liliana Durón Figueroa, Guillermo Hernández, Angélica González-Arrieta, Fernando de la Prieta

**Affiliations:** AIR Institute, IoT Digital Innovation Hub, Salamanca, Spain; BISITE Research Group, 16779 University of Salamanca, Salamanca, Spain; 16779 Institute of Biomedical Research of Salamanca (IBSAL), University of Salamanca, Salamanca, Spain; James Watt School of Engineering, University of Glasgow, Glasgow, UK

**Keywords:** deep learning, genomics, pathogenicity prediction, next-generation sequencing, variant classification, precision medicine

## Abstract

The rapid advancement of Next-Generation Sequencing (NGS) technologies has revolutionized the field of genomics, producing large volumes of data that necessitate sophisticated analytical techniques. This paper introduces a Deep Learning model designed to predict the pathogenicity of genetic variants, a vital component in advancing personalized medicine. The model is trained on a dataset derived from the analysis of NGS outputs, containing a combination of well-defined and ambiguous genetic variants. By employing a semi-supervised learning approach, the model efficiently utilizes both confidently labeled and less certain data. At the core of the methodology is the Feature Tokenizer Transformer architecture, which processes both numerical and categorical genomic information. The preprocessing pipeline includes key steps such as data imputation, scaling, and encoding to ensure high data quality. The results highlight the model’s impressive accuracy, particularly in detecting confidently labeled variants, while also addressing the impact of its predictions on less certain (soft-labeled) data.

## 1 Introduction

The field of genomics has experienced a dramatic transformation with the arrival of Next-Generation Sequencing (NGS) technologies [1], leading to a surge in the volume and complexity of genetic data available for research and clinical applications. This wealth of data presents both opportunities and challenges, particularly in the identification of pathogenic genetic variants associated with various diseases [2]. Effective analysis of such extensive datasets requires the development of advanced computational methods capable of distinguishing pathogenic variants from benign ones with high accuracy.

In recent years, machine learning (ML) and deep learning (DL) have emerged as powerful tools in genomics, offering the ability to automatically extract meaningful patterns from complex and high-dimensional data [3]. Traditional approaches for variant pathogenicity prediction often rely on fixed biological features, such as conservation scores or functional annotations, which may not fully capture the underlying complexities of genetic data [4]. In contrast, DL models, particularly those leveraging architectures like transformers, have shown promise in capturing intricate relationships within data, enhancing the predictive capabilities of these models [5].

This paper presents a novel deep learning model that leverages the Feature Tokenizer Transformer (FTT) architecture [6] to predict the pathogenicity of genetic variants. Our model is designed to integrate both numerical and categorical features from genomic data, utilizing a semi-supervised learning framework [7] to make the best use of labeled and unlabeled data. By employing a combination of supervised learning for well-defined cases and unsupervised techniques for ambiguous examples, the model aims to provide a more nuanced understanding of variant pathogenicity.

Furthermore, our approach addresses the significant challenge of label uncertainty in genomic datasets, particularly in the context of variants of uncertain significance (VUS). By refining the classification process into a binary format, we enhance the model’s ability to discern between benign and pathogenic variants while accounting for the probabilistic nature of the predictions. This capability is crucial for advancing personalized medicine and improving clinical decision-making based on genomic information.

In the following sections, we detail the methodology, including data preprocessing, model architecture, and training strategies, and present a thorough evaluation of the model’s performance. The results underscore the potential of this DL model in enhancing the accuracy of pathogenicity predictions, providing a foundation for further research and clinical applications.

This work is based on the initial findings presented at the 2024 PACBB conference [8].

## 2 State of the art

Predicting the pathogenicity of genomic variants has long been a central task in bioinformatics and clinical genetics. Over time, numerous methodologies have emerged, drawing on an expanding catalog of annotations that include evolutionary conservation, biochemical properties, and functional assays, as well as collective knowledge encoded in publicly available databases. The following section provides an overview of representative tools and their underlying principles, accompanied by a measured examination of commonly discussed limitations. Many of these tools are continually evolving, so updates to their software or databases may address some of the issues described below.

**ClinPred** [9] is a machine learning-based tool that employs random forest and gradient boosting models over features derived from dbNSFP and gnomAD. It specializes in missense variants, demonstrating strong predictive performance for protein-altering substitutions and outperforming other ensemble methods. ClinPred is widely used in clinical and research pipelines, though its performance on noncoding or splice-altering mutations is limited due to its training data focus on missense variants. Additionally, because it relies on periodically updated databases, older static versions may lack newly discovered variants.

**REVEL (Rare Exome Variant Ensemble Learner)** [10] integrates outputs from 13 functional prediction tools, including SIFT, PolyPhen-2, MutationTaster, and PROVEAN, within a random forest model. Optimized for rare missense variants, REVEL demonstrates superior performance in distinguishing pathogenic from benign substitutions. However, as an ensemble model, its interpretability can be affected by systematic biases or disagreements among its constituent predictors.

**MetaSVM** and **MetaLR** [11] are ensemble-based classifiers that aggregate multiple functional scores, utilizing a support vector machine (MetaSVM) and logistic regression (MetaLR) to enhance predictive sensitivity and specificity. These tools perform well for coding variants but remain limited for large insertions/deletions (indels) and deep intronic variants, which lack comprehensive feature representation.

**MAGPIE** [12] extends predictive capabilities to synonymous, nonsynonymous, and certain noncoding variants using a machine learning pipeline trained on ClinVar. MAGPIE integrates population frequency data, structural annotations, and additional genomic features, making it a versatile method for clinical variant interpretation. However, its performance depends on the completeness of reference databases, and frequent retraining may be necessary to adapt to newly discovered variants.

Ensemble-based classifiers like **VEST4** [13] and **M-CAP** [14] refine pathogenicity predictions by consolidating multiple computational scores. VEST4 prioritizes rare missense variants using supervised learning, while M-CAP employs gradient boosting to enhance discrimination of uncertain missense variants. Both tools demonstrate high accuracy but are limited by feature coverage variability across different genomic regions and populations.

Evolutionary conservation is central to predictors like **MutationAssessor** [15] and **PrimateAI** [16]. MutationAssessor derives functional impact from multiple sequence alignments, while PrimateAI utilizes primate-specific genomic data within a deep learning framework. Both methods excel at evaluating missense variants in highly conserved regions but provide limited insight into noncoding regions and structural variations.

Structure-based predictors, including **PolyPhen-2** [17], **SIFT4G** [18], and **LIST-S2** [19], leverage biochemical properties and protein structures to assess mutation effects. These tools are highly informative when reliable structural data exist but offer limited utility for variants in noncoding regions or within genes lacking high-resolution structural models.

Deep learning approaches, such as **DANN** [20], extend variant interpretation by encoding complex, nonlinear relationships across multiple genomic features. DANN improves classification for both coding and noncoding variants but requires extensive, well-labeled training data to prevent overfitting.

Overall, computational approaches have significantly improved the sensitivity and specificity of pathogenicity predictions, with many integrated into clinical workflows. However, persistent challenges remain, particularly in classifying non-missense variants, addressing the reliance on periodically updated databases, and resolving ambiguities in Variants of Uncertain Significance (VUS). The semi-supervised deep learning framework introduced in this paper seeks to address these challenges by systematically incorporating both well-labeled and uncertain data, broadening model coverage, and facilitating frequent updates as genomic knowledge expands.

## 3 Methodology

### 3.1 Dataset

The dataset employed to train the deep learning model was developed internally and is designed to reflect the typical format and content produced by advanced next-generation sequencing (NGS) analysis tools. The input data is in the form of a CSV file, derived from the annotation of a VCF file sourced from the ClinVar database, updated as of August 19, 2024. This file was processed using a custom-built bioinformatics pipeline. ClinVar, managed by the National Institutes of Health (NIH), compiles and curates information linking genetic variants to their clinical implications, drawing on submissions from research labs, clinical testing services, and expert review panels to provide comprehensive variant classifications and associated phenotypes [21].

#### 3.1.1 Genetic analysis pipeline

This pipeline, which processes Illumina [22] germline whole-exome sequencing data, operates under the Nextflow workflow management system [23]. It is designed to ensure parallel task execution, reproducible results, and compatibility with diverse computing environments. Its primary steps include:

– **Quality Control and Preprocessing:** Initial quality control is performed on the data to filter out low-quality reads, preparing it for deeper analysis.
– **Alignment:** The Burrows-Wheeler Aligner-Maximum Exact Match (bwa-mem) [24] algorithm is employed to align sequencing reads to a reference genome, locating genetic variants.
– **Variant Calling:** Advanced algorithms are used to identify a range of genetic variants, such as single nucleotide variants, insertions, deletions, and structural variants.
– **Annotation:** In this step, the functional impact of genetic variants is analyzed and contextualized within biological and clinical frameworks. The present analysis begins at this stage, utilizing the VCF file from ClinVar as input. Annotation was conducted with Ensembl Variant Effect Predictor (VEP) v.109 [25], OpenCravat v. 2.2.9 [26], and TAPES v. 0.1 [27], each contributing distinct insights into variant significance.

#### 3.1.2 Dataset composition

The resulting dataset is extensive, offering a thorough overview of the genomic data utilized for pathogenicity prediction. It comprises 376 columns, which are organized into six primary categories.

– **Basic Annotation:** Variants are documented with essential genomic details, such as gene names, chromosomal positions, and allelic variations.
– **Pathogenicity Predictors:** Computational tools integrated into VEP and OpenCravat assess the likelihood of a variant being pathogenic. These tools use various models and algorithms to evaluate the potential impact of variants on gene function and structure.
– **Population Allele Frequencies:** Data on the frequency of specific alleles in various populations is incorporated. This information helps determine the rarity of variants, which is crucial for assessing their potential pathogenicity.
– **Clinical Information:** Data linking genetic variations to clinical presentations, including patient symptoms and known disease associations, is included to evaluate the real-world impact of variants. This category is supported by information from ClinVar, which classifies variants based on clinical significance, expert reviews, and established guidelines.
– **Evolutionary Metrics:** Conservation of genetic regions across species is assessed to understand their biological importance. Tools that evaluate evolutionary conservation help in understanding the functional relevance of genetic regions and highlight potential pathogenic consequences of mutations.
– **Other Annotation Sources:** Additional insights are provided through integration with resources that offer valuable context on drug-gene interactions and literature-backed variant-disease associations.

The VEP utilizes a variety of plugins to analyze genetic variants. These plugins are designed to provide insights into different aspects of variant effects, such as pathogenicity, functional impact, evolutionary conservation, and gene function (refer to Table 1).

**Table 1:** Ensembl VEP plugins grouped by functionality.

Category	Plugin examples	Description
Pathogenicity and functional impact prediction	CADD [28], FATHMM [29], FATHMM_MKL [30], REVEL [10], LoF [31], LoFtool [32], SpliceAI [33]	Predicts variant impact and pathogenicity using multiple models.
Conservation and evolutionary constraint	Conservation [34], AncestralAllele, PrimateAI, G2P [35]	Evaluates conservation and predicts functional consequences.
Phenotypic and disease association	DisGeNET [36], Mastermind [37], phenotypes	Links variants to diseases and phenotypes.
Gene function and expression	LOEUF [31], GO [38], miRNA	Provides insights into gene function and expression.
Splicing and protein structure	MaxEntScan [39], GeneSplicer [40], ProteinSeqs	Analyzes impacts on splicing and protein structure.
Variant frequency and population	dbNSFP [41], gnomADc [42], Gwava	Provides allele frequencies and population-based predictions.
Miscellaneous	IntAct [43], NearestGene, LocalID	Additional context like protein interactions and local IDs.

OpenCravat employs several annotators to enhance the interpretation of genetic variants. These annotators are designed to provide detailed information about variant pathogenicity, clinical relevance, and population frequencies. Annotators focused on functional impact prediction evaluate the potential deleterious effects of variants, whereas those addressing clinical relevance link variants to known diseases and cancer mutations. Additionally, OpenCravat includes resources for population frequency data and additional context from various databases, which support the assessment of variant rarity and its implications (refer to Table 2).

**Table 2:** OpenCravat annotators grouped by functionality.

Category	Annotator examples	Description
Functional impact prediction	CScape coding [44], dbscSNV [45], ChasmPlus [46]	Assesses the impact of coding variants and splicing.
Clinical relevance and disease association	CIViC [47], COSMIC [48], cancer genome interpreter [49], CIViC gene	Links variants to diseases and cancer mutations.
Population frequency and GWAS	gnomAD gene, GWAS catalog [50], GRASP [51]	Provides allele frequencies and links to GWAS traits.
Conservation and evolutionary constraint	LINSIGHT [52], LOFtool, RVIS [53]	Scores conservation and mutation intolerance.
Gene and protein function	GO, NCBIGene [54], HG19	Provides insights into gene function and gene-related data.
Regulatory elements	Ensembl regulatory build [55], ESS gene	Annotates variants within regulatory elements.
Miscellaneous	dbSNP [56], MUPIT [57], LitVar [58]	Provides additional context including local IDs and variant effects.

In addition to the VEP and OpenCravat tools, which provide detailed insights into genetic variants through functional impact prediction and clinical relevance, TAPES is employed specifically for variant prioritization. TAPES uses American College of Medical Genetics and Genomics (ACMG) criteria [59] to systematically evaluate variants, focusing on distinguishing between those with pathogenic potential and those considered benign or of uncertain significance. The specific criteria are displayed in Figure 2.

#### 3.1.3 Data preprocessing

**Figure 1:**
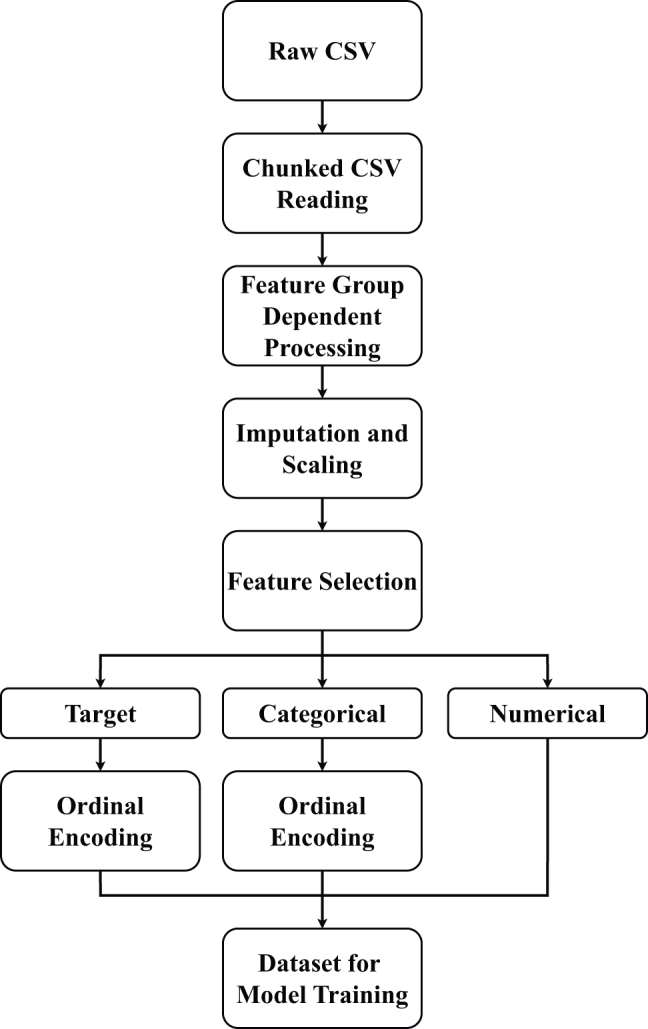
Data preprocessing pipeline.

The data preprocessing phase was essential in preparing the genomic data for our deep learning-based pathogenicity prediction model. The script used for this process applied multiple steps to ensure the data was well-suited for training (see Figure 1).

– **Loading Data:** Data was loaded from CSV files in chunks, using custom converters to enforce data types and manage missing values. This method facilitated efficient memory usage and allowed processing of large genomic datasets.
– **Feature Categorization and Processing:** Dataset features were grouped according to their type. Some features were one-hot encoded, others were binarized based on keyword presence, and specific features underwent custom processing depending on their characteristics.
– **Imputation and Scaling:** A heuristic approach was taken for imputation, filling missing values with worst-case assumptions when domain knowledge supported it. In cases without such assumptions, the mean or median was used based on the feature’s distribution. Features with more than 25 % missing values were excluded to maintain data reliability. For scaling, we applied standard scaling to normally distributed features, while min-max scaling was used for non-normally distributed features, ensuring values ranged between zero and one. This dual approach minimized biases and optimized the data for various analytical methods.
– **Feature Selection:** Features with significant missing data were discarded, and a variance threshold was applied to remove those with minimal variability, as such features are unlikely to enhance predictive performance. Given the dataset’s high dimensionality and the model’s ability to learn complex patterns, no additional feature selection was performed. The deep learning model is capable of handling large feature sets by identifying important patterns within the data. This strategy simplified preprocessing and allowed the model to leverage the full scope of available data, maximizing both information usage and predictive accuracy without compromising performance.
– **Categorical Encoding:** Categorical variables were encoded using an ordinal method, especially for features with inherent rankings, such as confidence levels and annotations. The target variable was numerically encoded based on ACMG guidelines.
– **Final Dataset Assembly:** The processed features, along with the encoded target variable, were consolidated to create the final dataset, fully prepared for model training. This preprocessing pipeline ensured the data was consistent, properly formatted, and optimized for the deep learning algorithms, ensuring that the model could efficiently learn from and generalize across the dataset.

**Figure 2:**
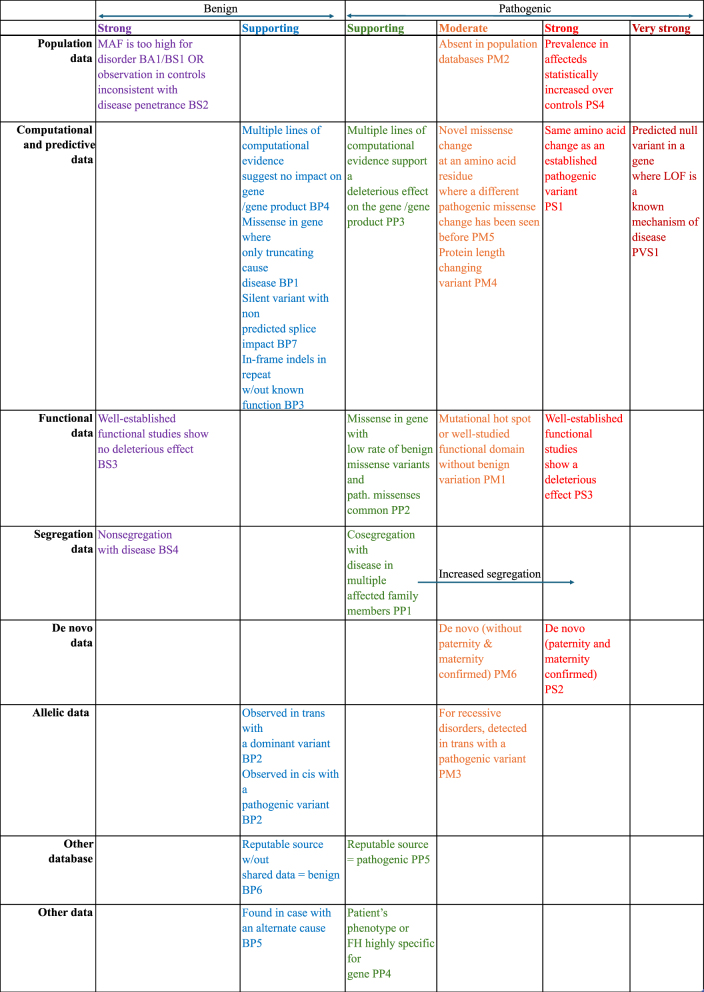
The chart categorizes each criterion by evidence type and strength for benign (left) or pathogenic (right) assertions. Abbreviations: BS (benign strong), BP (benign supporting), FH (family history), LOF (loss-of-function), MAF (minor allele frequency), path. (pathogenic), PM (pathogenic moderate), PP (pathogenic supporting), PS (pathogenic strong), PVS (pathogenic very strong). Extracted from [59].

### 3.2 Classification target exploration

In the dataset, six unique labels are defined according to the ACMG guidelines, covering a spectrum of pathogenicity and certainty in classification. These categories are: ”Benign auto”, ”Benign”, ”Likely Benign”, ”Likely Pathogenic”, ”Pathogenic”, and ”VUS” (Variant of Uncertain Significance). Each of these categories corresponds to specific criteria combinations based on the ACMG framework.

– **Benign auto**: This label refers to variants automatically classified as benign based on the strongest evidence. The designation stems from the presence of the BA1 criterion, which indicates a very high allele frequency in control populations, demonstrating that the variant is too common to cause a rare genetic disorder. This criterion is often applied as an exclusionary filter, meaning that if a variant meets BA1, it can be classified as benign without needing to assess additional evidence [60].
– **Benign**: Variants classified as ”Benign” meet stringent evidence of non-pathogenicity. In our dataset, a variant is labeled ”Benign” if it satisfies at least two BS (Benign Strong) criteria, such as observation in a healthy individual without disease or computational evidence suggesting no functional impact.
– **Likely Benign**: A ”Likely Benign” label is assigned when the evidence leans towards non-pathogenicity but is not definitive. This classification is applied when at least one BS criterion is met alongside BP (Benign Supporting) criteria, which are weaker lines of evidence, or if two BP criteria are satisfied. These variants are considered unlikely to be disease-causing but do not meet the stringent evidence required for a full benign classification.
– **Pathogenic**: This label denotes variants with strong evidence of pathogenicity. A variant is classified as ”Pathogenic” when it satisfies the PVS1 criterion (a null variant in a gene where loss of function is a known disease mechanism), in combination with other strong (PS), moderate (PM), or supporting (PP) pathogenicity criteria. Such variants are highly likely to be disease-causing.
– **Likely Pathogenic**: ”Likely Pathogenic” variants have significant evidence pointing towards pathogenicity but fall short of the certainty required for a definitive pathogenic classification. This label is applied when a variant meets moderate (PM) and supporting (PP) criteria or has a PVS1 variant combined with other weaker evidence. While these variants are considered to be disease-causing, the evidence is not as conclusive as for the ”Pathogenic” category.
– **VUS**: These variants fall into an ambiguous category where the available evidence is insufficient to determine whether they are pathogenic or benign. These variants do not meet the criteria for any of the other categories, often due to a lack of data or conflicting evidence regarding their impact.

However, this setup is not ideal for machine learning targets, as it mixes pathogenicity with classification certainty. To refine this, we aim to focus solely on pathogenicity, allowing the inherent probabilistic characteristics of ML algorithms to account for classification uncertainty.

To achieve this, we convert the problem into a binary classification task, where the model predicts either a benign or a pathogenic outcome. We achieve this binarization by grouping the six initial labels into two categories: *hard labels*–comprising *Benign auto*, *Benign*, and *Pathogenic*; and *soft labels*–including *Likely Benign*, *Variant of Uncertain Significance (VUS)*, and *Likely Pathogenic*. It is important to understand that the majority of our dataset is softly labeled, with over 80 % of the data classified as VUS and only about 2 % having definitive, hard labels. This imbalance in label distribution underscores the need for a semi-supervised learning approach to effectively leverage the available data.

## 4 Architecture and training

**Figure 3:**
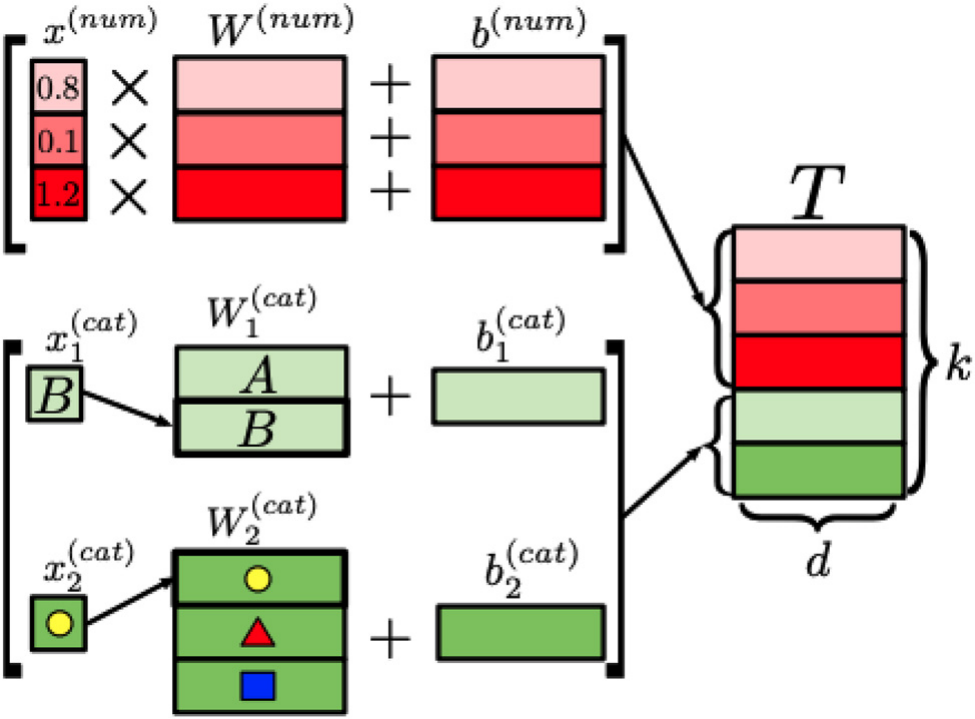
Illustration of feature tokenization within the FTT architecture for *k* features and latent dimension *d*. Adapted from [6].

The model architecture chosen for our pathogenicity predictor is the FTT [6]. This selection was driven by the necessity to effectively handle both numerical and categorical features in our dataset, a key strength of the FTT architecture. In an FTT, after all features are properly encoded, they are tokenized using an embedding layer within the model, as depicted in Figure 3.

This embedding or tokenization process converts features into dense vectors of a predetermined size, allowing the model to capture more intricate patterns in the data. In the context of genomic data, this method is particularly advantageous for handling features such as gene sequences or categorical variables with a large number of unique values, as it enables the model to develop a more nuanced understanding of the data.

**Figure 4:**
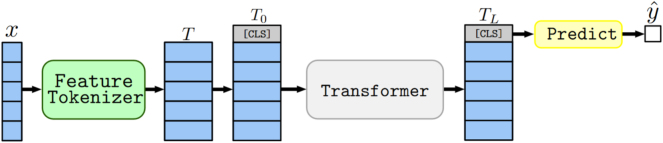
Summary of FTT architecture. Extracted from [6].

A dedicated ‘classification’ token ([CLS]) is then added at the beginning of the latent representation for each sample. This is a standard technique when utilizing transformers for classification tasks (as seen in [61]). The stack of tokens created through this process is passed through the model’s multiple transformer layers. Ultimately, the transformed [CLS] token is fed into a classification head, which consists of a simple linear layer that outputs the class probabilities. The overall architecture is summarized in Figure 4.

Before feeding the data to the DL model for the training process and the subsequent evaluations, it was split with a 70/15/15 ratio between training, validation and testing datasets. This split was stratified to maintain the class imbalance consistent across the three splittings of the dataset.

### 4.1 Semi-supervised training

A semi-supervised training approach was used to train the model to maximize the utility of softly labeled data, which might otherwise be underutilized or misinterpreted. We employed a straightforward pseudo-labeling technique as proposed in [62], using equal weights for both labeled and pseudo-labeled losses. This method was chosen over more recent approaches (such as consistency regularization [63], 64]) due to its simplicity and, crucially, to avoid introducing synthetic or augmented data, which may raise concerns about interpretability, particularly within the medical community.

Initially, the model is trained using only the hard-labeled samples with binary outcomes, as described previously. After convergence, the trained model is used in inference mode to predict the classes of softly labeled samples. Those samples with a prediction confidence score exceeding a specified threshold are added to the training set with their predicted labels. The model is then retrained with this expanded dataset. This process is repeated until no additional samples meet the confidence threshold or a predetermined maximum number of iterations is reached. In our setup, we used a confidence threshold of 95 % and allowed up to 10 iterations.

At the end of the semi-supervised iterations, only a small number of Likely Benign and Likely Pathogenic cases did not reach the confidence threshold and hence were not included in the training and validation data. The fraction that were not included in the data fed to the model was in all cases below the 1 % level for each label and data split.

For optimization, we employed the Adam algorithm with weight decay [65]. To further enhance generalization and mitigate overfitting, we incorporated several standard regularization techniques during training, most notably dropout and label smoothing. Dropout [66], 67] involves randomly disabling a portion of a layer’s output to prevent overfitting, and it was applied to the attention, feed-forward, and residual layers within the transformer blocks. Label smoothing [68], 69] adds uniform noise to the labels, accounting for potential labeling errors and enhancing model generalization–a critical aspect in our setup, given the likelihood of misclassifying some softly labeled samples during the semi-supervised process.

Regarding hyperparameters, the token dimension was set to 64, with three stacked transformer layers, each containing four heads in the multi-headed attention sublayers and a hidden dimension of 456 in their feed-forward sublayers. Dropout rates were configured at 0.1 for all attention layers, feed-forward layers and residual connections. A gated Gaussian error linear unit (GELU) was used as activation function for the non-linearity. We opted for prenormalization instead of postnormalization, favoring ease of optimization over peak performance [6]. For the semi-supervised parameters, the confidence threshold was set at 95 %, and a maximum of 10 iterations was allowed. The Adam optimizer had a learning rate of 10^−4^ with a weight decay of 10^−5^. Finally, the label smoothing parameter was set to 0.05.

## 5 Results and discussion

We developed a DL model to predict the pathogenicity of genetic variants, and its effectiveness was evaluated using a confusion matrix. The analysis considered the six specified labels, and aimed to distinguish between benign and pathogenic variants through a binary classification approach (refer to Table 3).

**Table 3:** Confusion matrix on test set (original labels against hard predicted labels).

		Predicted label	
		Benign	Pathogenic
Original label	Benign auto	883	1
	Benign	17	0
	Likely benign	27,962	142
	VUS	266,535	79,460
	Likely pathogenic	4,691	11,633
	Pathogenic	0	6,079

**Figure 5:**
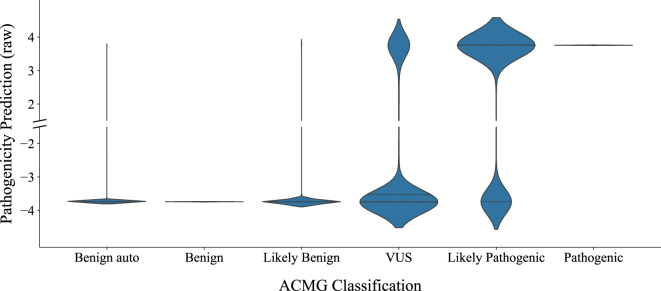
Violin plot showing the distribution of predictions, before applying sigmoid function, for each target label in the dataset.

We also extracted the distribution of predictions for each target label before applying the final sigmoid layer used to binarize the output of the model. This is shown in Figure 5. The plot shows the great confidence the model has in its predictions, with only a negligible number of predictions lying in the interval between −2 and 2. This has no medical implications, but establishes clear predictions to test in future works.

– The model demonstrated strong accuracy in correctly identifying hard labels, as shown by its results with ‘Benign auto’, ‘Benign’, and ‘Pathogenic’ categories. Accurately predicting ‘Pathogenic’ variants is particularly crucial due to the significant implications of misclassification.
– For ‘VUS’, the model applied a sophisticated classification method, indicating its potential effectiveness in identifying pathogenic variants in uncertain cases.
– The handling of ‘Likely Pathogenic’, ‘VUS’, and ‘Likely Benign’ variants appears to effectively simplify the soft label challenge into a binary format. However, due to the inherent uncertainty in these labels, additional study is necessary to refine their interpretation.
– The strong confidence of the model predictions provides a great basis for testing when more hard-labeled data is publicly available.

Beyond these technical observations, there are potential implications for clinical practice. In particular, the semi-supervised approach could aid in reclassifying VUS by identifying those most likely to be pathogenic, thus guiding more focused laboratory investigations or functional studies. Likewise, the ability to integrate uncertain samples may eventually help genomics laboratories or clinical testing facilities reduce the time spent on manual curation, provided that large-scale prospective validations confirm the reliability of these semi-supervised predictions. However, because the data in this study derive primarily from public repositories without longitudinal follow-up, we do not claim immediate applicability for clinical diagnostics. Future studies that incorporate patient-level outcomes, cross-reference the model’s predictions with established clinical reports, and conduct experimental assays on borderline cases will be essential steps toward realizing this framework’s practical utility in genomic medicine.

We have performed a direct comparison of our method with ClinPred [9] and REVEL [10], both based on decision trees (Table 4). Our method achieves higher specificity and precision, at the cost of lower sensitivity and accuracy. This is due to the large number of “false negatives” predicted by our model (see Likely Pathogenic variants in Table 3). However, it is important to highlight a key difference between our approach and the one in [9]. While they consider some soft-labeled variants (Likely Pathogenic and Likely Benign) as ground truth, we handle them differently, classifying them dynamically as seen in 4.1. Rather than assuming certainty in these labels, our semi-supervised approach allows the model to learn from them adaptively, refining predictions based on broader patterns in the data. This not only reduces the risk of propagating label noise but also enhances the model’s ability to generalize to novel variants with uncertain classifications. Future work could compare the performance of ClinPred under a similar semi-supervised setup, which would provide valuable insights into the trade-offs between precision and recall in pathogenicity prediction.

**Table 4:** Comparison between different methods. With data extracted from [9]. Abbreviations: FPR, false positive rate; MCC, matthews correlation coefficient.

	Sensitivity %	Specificity %	FPR	Accuracy	Precision	F1 score	MCC	
FTT (ours)	79.06	**99.51**	**0.005**	0.91	**0.99**	0.88	0.82	
ClinPred	**93.58**	94.10	0.060	**0.94**	0.86	**0.90**	**0.85**	
REVEL	82.55	89.27	0.110	0.87	0.75	0.78	0.70	

The bold values represents the best-performing score for each metric across the three methods.

## 6 Conclusions

This study introduced a DL model designed to predict the pathogenicity of genetic variants, a crucial advancement for personalized medicine and the integration of genomics into clinical practice. The model employs a semi-supervised learning strategy combined with the FTT architecture to manage the inherent complexities of genomic data. Our results indicate that the model can effectively classify genetic variants ranging from benign to clearly pathogenic, although the full implications of the soft labels remain somewhat ambiguous.

Although the initial findings are encouraging, there are still areas for potential improvement. Future research will aim to enhance the model by expanding the training dataset, incorporating a wider array of genomic features, and improving its ability to detect subtle genetic variations. Moreover, continued validation through functional studies and clinical correlations will be necessary to ensure the model’s applicability and reliability in real-world clinical environments.

## Appendix A: Dataset variable descriptions

This appendix provides a detailed list of the variables included in the dataset used for pathogenicity prediction. (Table A1).

**Table A1:** Dataset description.

Variable name	Description
Chromosome (hg38)	Chromosome (hg38)
Position (hg38)	Position (hg38)
Reference allele	Reference allele
Alternative allele	Alternative allele
Gene (HUGO)	Gene name by HUGO
MANE SELECT	The MANE Select set consists of one transcript at each locus across the genome that is representative of biology at that locus.
MANE PLUS CLINICAL	The MANE plus clinical set includes additional transcripts for genes where MANE select alone is not sufficient to report all ‘pathogenic (P)’ or ‘likely pathogenic (LP)’ clinical variants available in public resources.
HGVSc	HGVS coding variant presentation from VEP
HGVSp	HGVS protein variant presentation from VEP
HGVSg	HGVS genomic variant presentation from VEP
Probability path	Pathogenicity probability score by TAPES
Prediction ACMG	Prediction by American College of medical genetics and genomics (ACMG)
Start position	Start position
End position	End position
Chromosome (hg19)	Chromosome (hg19)
Position (hg19)	Position (hg19)
Transcript	Transcript ID by ensembl
Sequence Ontology	Variant consequence by SO
cDNA change	cDNA change
Protein change	Protein change
All mappings	All mapping transcripts
Ensembl gene ID	Gene ID by ensembl
Ensembl feature ID	Feature ID by ensembl
Feature type	Feature type
CDS strand	Coding sequence (CDS) strand (+ or -)
cDNA position	Relative position of base pair in cDNA sequence
CDS position	Relative position of base pair in coding sequence
Protein position	Relative position of base pair in protein
Aminoacids	Amino acids involved in the variant
Codons	Codons involved in the variant
Impact	Impact of variant predicted by ensembl
Distance	Distance to the nearest gene
Strand	Indicates the strand where the variant is located, + (1) or - (−1)
HGNC ID	HUGO gene nomenclature committee ID
TSL	Transcript support level
APPRIS	APPRIS isoform annotation
CCDS	CCDS id
ENSP	Ensembl ENSP id
Exon	Exon number
Intron	Intron number
Clinical significance	ClinVar clinical significance
Pubmed	PubMed publications identifiers
1000Gp3 AC	Alternative allele counts in the whole 1000 genomes phase 3 (1000Gp3) data
1000Gp3 AF	Alternative allele frequency in the whole 1000Gp3 data
ALSPAC AC	Alternative allele count in called genotypes in UK10K ALSPAC cohort
ALSPAC AF	Alternative allele frequency in called genotypes in UK10K ALSPAC cohort
Aloft confidence	Confidence level of Aloft pred. Values can be ‘high confidence’ (*p* < 0.05) or ‘low confidence’ (*p* > 0.05)
Aloft fraction transcripts affected	The fraction of the transcripts of the gene affected i.e. No. of transcripts affected by the SNP/Total no. of protein coding transcripts for the gene
Aloft prediction	Final classification predicted by ALoFT. Values can be Tolerant, Recessive or Dominant
Aloft Probability Dominant	Probability of the SNP being classified as dominant disease-causing by ALoFT
Aloft Probability Recessive	Probability of the SNP being classified as recessive disease-causing by ALoFT
Aloft Probability Tolerant	Probability of the SNP being classified as benign by ALoFT
AltaiNeandertal	Genotype of a deep sequenced Altai Neanderthal
Ancestral allele	Ancestral allele based on 8 primates EPO. Ancestral alleles by ensembl 84. The following comes from its original README file: ACTG – high-confidence call, ancestral state supported by the other two sequences actg – low-confidence call, ancestral state supported by one sequence only N–failure, the ancestral state is not supported by any other sequence – the extant species contains an insertion at this position. – no coverage in the alignment
BayesDel addAF prediction	Prediction of BayesDel addAF score based on the authors’ recommendation, ‘T(olerated)’ or ‘D(amaging)’. The score cutoff between ‘D’ and ‘T’ is 0.0692655
BayesDel addAF rankscore	The rankscore is the ratio of the rank of the score over the total number of BayesDel_addAF scores in dbNSFP
BayesDel addAF score	A deleteriousness preidction meta-score for SNVs and indels with inclusion of MaxAF. The higher the score, the more likely the variant is pathogenic. The author suggested cutoff between deleterious (‘D’) and tolerated (‘T’) is 0.0692655
BayesDel noAF prediction	Prediction of BayesDel noAF score based on the authors’ recommendation, ‘T(olerated)’ or ‘D(amaging)’. The score cutoff between ‘D’ and ‘T’ is −0.0570105
BayesDel noAF rankscore	The rankscore is the ratio of the rank of the score over the total number of BayesDel_noAF scores in dbNSFP
BayesDel noAF score	A deleteriousness preidction meta-score for SNVs and indels without inclusion of MaxAF. The higher the score, the more likely the variant is pathogenic. The author suggested cutoff between deleterious (‘D’) and tolerated (‘T’) is −0.0570105
DANN rankscore	The rankscore is the ratio of the rank of the score over the total number of DANN scores in dbNSFP
DANN score	DANN is a functional prediction score retrained based on the training data of CADD using deep neural network. Scores range from 0 to 1. A larger number indicate a higher probability to be damaging
DEOGEN2 prediction	Prediction of DEOGEN2 score based on the authors’ recommendation, ‘T(olerated)’ or ‘D(amaging)’. The score cutoff between ‘D’ and ‘T’ is 0.5
DEOGEN2 rankscore	The rankscore is the ratio of the rank of the score over the total number of DEOGEN2 scores in dbNSFP
DEOGEN2 score	A deleteriousness prediction score ‘which incorporates heterogeneous information about the molecular effects of the variants, the domains involved, the relevance of the gene and the interactions in which it participates’. It ranges from 0 to 1. The larger the score, the more likely the variant is deleterious. The authors suggest a threshold of 0.5 for separating damaging vs tolerant variants
Denisova	Genotype of a deep sequenced Denisova
Eigen-PC-Phred coding	Eigen PC score in phred scale
Eigen-PC-raw coding	Eigen PC score for genome-wide SNVs. A functional prediction score based on conservation, allele frequencies, deleteriousness prediction (for missense SNVs) and epigenomic signals (for synonymous and non-coding SNVs) using an unsupervised learning method
Eigen-PC-raw coding rankscore	The rankscore is the ratio of the rank of the score over the total number of eigen-PC-raw scores in dbNSFP
Eigen-Phred coding	Eigen score in phred scale
Eigen-raw coding	Eigen score for coding SNVs. A functional prediction score based on conservation, allele frequencies, and deleteriousness prediction using an unsupervised learning method
Eigen-raw coding rankscore	The rankscore is the ratio of the rank of the score over the total number of eigen-raw scores in dbNSFP
FATHMM converted rankscore	FATHMM scores were first converted to FATHMMnew = 1-(FATHMMori+16.13)/26.77, then ranked among all FATHMMnew scores in dbNSFP. The rankscore is the ratio of the rank of the score over the total number of FATHMMnew scores in dbNSFP. If there are multiple scores, only the most damaging (largest) rankscore is presented. The scores range from 0 to 1
FATHMM prediction	If a FATHMM score is <=−1.5 the corresponding nsSNV is predicted as ‘D(AMAGING)’; otherwise it is predicted as ‘T(OLERATED)’
FATHMM score	FATHMM default score (weighted for human inherited-disease mutations with disease Ontology). Scores range from −16.13 to 10.64. The smaller the score the more likely the SNP has damaging effect
GERP++ NR	GERP++ neutral rate
GERP++ RS	GERP++ RS score, the larger the score, the more conserved the site. Scores range from −12.3 to 6.17
GERP++ RS rankscore	The rankscore is the ratio of the rank of the score over the total number of GERP++ RS scores in dbNSFP
GM12878 confidence value	0 - highly significant scores (approx. *p* < 0.003); 1 – significant scores (approx. *p* < 0.05); 2 – informative scores (approx. *p* < 0.25); 3 – other scores (approx. *p* > = 0.25)
GM12878 fitCons rankscore	The rankscore is the ratio of the rank of the score over the total number of GM12878 fitCons scores in dbNSFP
GM12878 fitCons score	fitCons score predicts the fraction of genomic positions belonging to a specific function class (defined by epigenomic ‘fingerprint’) that are under selective pressure. Scores range from 0 to 1, with a larger score indicating a higher proportion of nucleic sites of the functional class the genomic position belong to are under selective pressure, therefore more likely to be functional important. GM12878 fitCons scores are based on cell type GM12878
GTEx V8 gene	Target gene of the (significant) eQTL SNP
GTEx V8 tissue	Tissue type of the expression data with which the eQTL/gene pair is detected
H1-hESC confidence value	0 - highly significant scores (approx. *p* < 0.003); 1 – significant scores (approx. *p* < 0.05); 2 – informative scores (approx. *p* < 0.25); 3 – other scores (approx. *p* > = 0.25)
H1-hESC fitCons rankscore	The rankscore is the ratio of the rank of the score over the total number of H1-hESC fitCons scores in dbNSFP
H1-hESC fitCons score	fitCons score predicts the fraction of genomic positions belonging to a specific function class (defined by epigenomic ‘fingerprint’) that are under selective pressure. Scores range from 0 to 1, with a larger score indicating a higher proportion of nucleic sites of the functional class the genomic position belong to are under selective pressure, therefore more likely to be functional important. GM12878 fitCons scores are based on cell type H1-hESC
HUVEC confidence value	0 - highly significant scores (approx. *p* < 0.003); 1 – significant scores (approx. *p* < 0.05); 2 – informative scores (approx. *p* < 0.25); 3 – other scores (approx. *p* > = 0.25)
HUVEC fitCons rankscore	The rankscore is the ratio of the rank of the score over the total number of HUVEC fitCons scores in dbNSFP
HUVEC fitCons score	fitCons score predicts the fraction of genomic positions belonging to a specific function class (defined by epigenomic ‘fingerprint’) that are under selective pressure. Scores range from 0 to 1, with a larger score indicating a higher proportion of nucleic sites of the functional class the genomic position belong to are under selective pressure, therefore more likely to be functional important. GM12878 fitCons scores are based on cell type HUVEC
Interpro domain	Domain or conserved site on which the variant locates. Domain annotations come from interpro database. The number in the brackets following a specific domain is the count of times interpro assigns the variant position to that domain, typically coming from different predicting databases. Multiple entries separated by ‘;’
LIST-S2 prediction	Prediction of LIST-S2 score based on the authors’ recommendation, ‘T(olerated)’ or ‘D(amaging)’. The score cutoff between ‘D’ and ‘T’ is 0.85
LIST-S2 rankscore	The rankscore is the ratio of the rank of the score over the total number of LIST-S2 scores in dbNSFP
LIST-S2 score	A deleteriousness preidction score for nonsynonymous SNVs. The range of the score in dbNSFP is from 0 to 1. The higher the score, the more likely the variant is pathogenic. The author suggested cutoff between deleterious (‘D’) and tolerated (‘T’) is 0.85
LRT Omega	Estimated nonsynonymous-to-synonymous-rate ratio (Omega, reported by LRT)
LRT converted rankscore	LRTori scores were first converted as LRTnew = 1-LRTori*0.5 if *Omega* < 1, or LRTnew = LRTori*0.5 if *Omega* > = 1. Then LRTnew scores were ranked among all LRTnew scores in dbNSFP. The rankscore is the ratio of the rank over the total number of the scores in dbNSFP. The scores range from 0.00162 to 0.8433
LRT prediction	LRT prediction, D(eleterious), N(eutral) or U(nknown), which is not solely determined by the score.
LRT score	The original LRT two-sided p-value (LRTori), ranges from 0 to 1
M-CAP prediction	Prediction of M-CAP score based on the authors’ recommendation, ‘T(olerated)’ or ‘D(amaging)’. The score cutoff between ‘D’ and ‘T’ is 0.025
M-CAP rankscore	The rankscore is the ratio of the rank of the score over the total number of M-CAP scores in dbNSFP
M-CAP score	M-CAP is a hybrid ensemble score. Scores range from 0 to 1. The larger the score the more likely the SNP has damaging effect.
MPC rankscore	The rankscore is the ratio of the rank of the score over the total number of MPC scores in dbNSFP
MPC score	A deleteriousness prediction score for missense variants based on regional missense constraint. The range of MPC score is 0–5. The larger the score, the more likely the variant is pathogenic
MVP rankscore	The rankscore is the ratio of the rank of the score over the total number of MVP scores in dbNSFP
MVP score	A pathogenicity prediction score for missense variants using deep learning approach. The range of MVP score is from 0 to 1. The larger the score, the more likely the variant is pathogenic. The authors suggest thresholds of 0.7 and 0.75 for separating damaging vs tolerant variants in constrained genes (*ExACpLI* > = 0.5) and non-constrained genes (*ExACpLI* < 0.5), respectively.
MetaLR prediction	Prediction of MetaLR based ensemble prediction score,‘T(olerated)’ or ‘D(amaging)’. The score cutoff between ‘D’ and ‘T’ is 0.5
MetaLR rankscore	The rankscore is the ratio of the rank of the score over the total number of MetaLR scores in dbNSFP. The scores range from 0 to 1
MetaLR score	Logistic regression (LR) based ensemble prediction score, which incorporated 10 scores (SIFT, PolyPhen-2 HDIV, PolyPhen-2 HVAR, GERP++, MutationTaster, mutation Assessor, FATHMM, LRT, SiPhy, PhyloP) and the maximum frequency observed in the 1000 genomes populations. Larger value means the SNV is more likely to be damaging. Scores range from 0 to 1
MetaSVM prediction	Prediction of SVM based ensemble prediction score,‘T(olerated)’ or ‘D(amaging)’. The score cutoff between ‘D’ and ‘T’ is 0
MetaSVM rankscore	The rankscore is the ratio of the rank of the score over the total number of MetaSVM scores in dbNSFP. The scores range from 0 to 1
MetaSVM score	Support vector machine (SVM) based ensemble prediction score, which incorporated 10 scores (SIFT, PolyPhen-2 HDIV, PolyPhen-2 HVAR, GERP++, MutationTaster, mutation Assessor, FATHMM, LRT, SiPhy, PhyloP) and the maximum frequency observed in the 1000 genomes populations. Larger value means the SNV is more likely to be damaging.
MutPred protID	UniProt accession or ensembl transcript ID used for MutPred score calculation
MutPred rankscore	The rankscore is the ratio of the rank of the score over the total number of MutPred scores in dbNSFP
MutPred score	General MutPred score. Scores range from 0 to 1. The larger the score the more likely the SNP has damaging effect
MutationAssessor prediction	MutationAssessor’s functional impact of a variant–predicted functional, i.e. high (‘H’) or medium (‘M’), or predicted non-functional, i.e. low (‘L’) or neutral (‘N’). The score cutoffs between ‘H’ and ‘M’, ‘M’ and ‘L’, and ‘L’ and ‘N’, are 3.5, 1.935 and 0.8, respectively
MutationAssessor rankscore	The rankscore is the ratio of the rank of the score over the total number of MAori scores in dbNSFP. The scores range from 0 to 1
MutationAssessor score	MutationAssessor functional impact combined score
MutationTaster prediction	MutationTaster prediction, ‘A’ (‘disease_causing_automatic’), ‘D’ (‘disease_causing’), ‘N’ (’polymorphism’) or ‘P’ (‘polymorphism_automatic’)
MutationTaster converted rankscore	The MTori scores were first converted. If the prediction is ‘A’ or ‘D’ MTnew = MTori; if the prediction is ‘N’ or ‘P’, MTnew = 1-MTori. Then MTnew scores were ranked among all MTnew scores in dbNSFP. If there are multiple scores of an SNV, only the largest MTnew was used in ranking. The rankscore is the ratio of the rank of the score over the total number of MTnew scores in dbNSFP. The scores range from 0.08979 to 0.81001.
MutationTaster score	MutationTaster p-value, ranges from 0 to 1.
PROVEAN prediction	If PROVEAN <=−2.5 the corresponding nsSNV is predicted as ‘D(amaging)’; otherwise it is predicted as ‘N(eutral)’.
PROVEAN converted rankscore	PROVEANori were first converted to *PROVEANnew* = 1 − (*PROVEANori* + 14)/28, then ranked among all PROVEANnew scores in dbNSFP. The rankscore is the ratio of the rank the PROVEANnew score over the total number of PROVEANnew scores in dbNSFP. If there are multiple scores, only the most damaging (largest) rankscore is presented. The scores range from 0 to 1
PROVEAN score	Scores range from −14 to 14. The smaller the score the more likely the SNP has damaging effect.
PrimateAI prediction	Prediction of PrimateAI score based on the authors’ recommendation, ‘T(olerated)’ or ‘D(amaging)’. The score cutoff between ‘D’ and ‘T’ is 0.803
PrimateAI rankscore	The rankscore is the ratio of the rank of the score over the total number of PrimateAI scores in dbNSFP
PrimateAI score	A pathogenicity prediction score for missense variants based on common variants of non-human primate species using a deep neural network. The range of PrimateAI score is 0–1. The larger the score, the more likely the variant is pathogenic
Reliability index	Number of observed component scores (except the maximum frequency in the 1000 genomes populations) for MetaSVM and MetaLR. Ranges from 1 to 10. As MetaSVM and MetaLR scores are calculated based on imputed data, the less missing component scores, the higher the reliability of the scores and predictions.
SIFT4G prediction	If SIFT4G is <0.05 the corresponding nsSNV is predicted as ‘D(amaging)’; otherwise it is predicted as ‘T(olerated)’.
SIFT4G converted rankscore	SIFT4G scores were first converted to SIFT4Gnew = 1-SIFT4G, then ranked among all SIFT4Gnew scores in dbNSFP. The rankscore is the ratio of the rank the SIFT4Gnew score over the total number of SIFT4Gnew scores in dbNSFP. If there are multiple scores, only the most damaging (largest) rankscore is presented
SIFT4G score	Scores range from 0 to 1. The smaller the score the more likely the SNP has damaging effect
SIFT prediction	If SIFT is smaller than 0.05 the corresponding nsSNV is predicted as ‘D(amaging)’; otherwise it is predicted as ‘T(olerated)’.
SIFT converted rankscore	SIFTori scores were first converted to SIFTnew = 1-SIFTori, then ranked among all SIFTnew scores in dbNSFP. The rankscore is the ratio of the rank the SIFTnew score over the total number of SIFTnew scores in dbNSFP. If there are multiple scores, only the most damaging (largest) rankscore is presented. The rankscores range from 0.00964 to 0.91255
SIFT score	Scores range from 0 to 1. The smaller the score the more likely the SNP has damaging effect
SiPhy 29way logOdds	SiPhy score based on 29 mammals genomes. The larger the score, the more conserved the site
SiPhy 29way logOdds rankscore	The rankscore is the ratio of the rank of the score over the total number of SiPhy_29way_logOdds scores in dbNSFP
SiPhy 29way pi	The estimated stationary distribution of A, C, G and T at the site, using SiPhy algorithm based on 29 mammals genomes.
Uniprot ACC	Uniprot accession number
Uniprot entry	Uniprot entry ID
VindijiaNeandertal	Genotype of a deep sequenced Vindijia Neandertal
bStatistic	Background selection (B) value estimates. Ranges from 0 to 1000. It estimates the expected fraction (*1000) of neutral diversity present at a site. Values close to 0 represent near complete removal of diversity as a result of background selection and values near 1000 indicating absent of background selection
bStatistic converted rankscore	bStatistic scores were first converted to -bStatistic, then ranked among all -bStatistic scores in dbNSFP. The rankscore is the ratio of the rank of -bStatistic over the total number of -bStatistic scores in dbNSFP
ClinVar MedGen ID	MedGen ID from ClinVar
ClinVar OMIM ID	OMIM ID from ClinVar
ClinVar Orphanet ID	Orphanet ID from ClinVar
ClinVar HGVS	HGVS nomenclature from ClinVar
ClinVar ID	ClinVar ID
ClinVar review	Review status of variant from ClinVar
ClinVar Trait	Trait associated with variant according to ClinVar
Fathmm-MKL coding group	The groups of features (labeled A-J) used to obtained the score
Fathmm-MKL coding prediction	If a fathmm-MKL coding score is >0.5 the corresponding nsSNV is predicted as ‘D(AMAGING)’; otherwise it is predicted as ‘N(EUTRAL)’
Fathmm-MKL coding rankscore	The rankscore is the ratio of the rank of the score over the total number of fathmm-MKL coding scores in dbNSFP
Fathmm-MKL coding score	Fathmm-MKL p-values. Scores range from 0 to 1. SNVs with scores >0.5 are predicted to be deleterious, and those <0.5 are predicted to be neutral or benign. Scores close to 0 or 1 are with the highest-confidence. Coding scores are trained using 10 groups of features
Fathmm-XF coding prediction	If a fathmm-XF coding score is >0.5 , the corresponding nsSNV is predicted as ‘D(AMAGING)’; otherwise it is predicted as ‘N(EUTRAL)’
Fathmm-XF coding rankscore	The rankscore is the ratio of the rank of the score over the total number of fathmm-XF coding scores in dbNSFP
Fathmm-XF coding score	Fathmm-XF p-values. Scores range from 0 to 1. SNVs with scores >0.5 are predicted to be deleterious, and those <0.5 are predicted to be neutral or benign. Scores close to 0 or 1 are with the highest-confidence. Coding scores are trained using 10 groups of features
gnomAD exomes AC	Alternative allele count in the whole gnomAD exome samples (125,748 samples)
gnomAD exomes AF	Alternative allele frequency in the whole gnomAD exome samples (125,748 samples)
gnomAD exomes AN	Total allele count in the whole gnomAD exome samples (125,748 samples)
gnomAD exomes POPMAX AC	Allele count in the population with the maximum AF
gnomAD exomes POPMAX AF	Maximum allele frequency across populations (excluding samples of Ashkenazi, Finnish, and indeterminate ancestry)
gnomAD exomes POPMAX AN	Total number of alleles in the population with the maximum AF
gnomAD exomes flag	Information from gnomAD exome data indicating whether the variant falling within low-complexity (lcr) or segmental duplication (segdup) or decoy regions. The flag can be either. For high-quality PASS or not reported/polymorphic in gnomAD exomes, ‘lcr’ for within lcr, ‘segdup’ for within segdup, or ‘decoy’ for with decoy region
gnomAD genomes AC	Alternative allele count in the whole gnomAD genome samples (71,702 samples)
gnomAD genomes AF	Alternative allele frequency in the whole gnomAD genome samples (71,702 samples)
gnomAD genomes AN	Total allele count in the whole gnomAD genome samples (71,702 samples)
gnomAD genomes POPMAX AC	Allele count in the population with the maximum AF
gnomAD genomes POPMAX AF	Maximum allele frequency across populations (excluding samples of Ashkenazi, Finnish, and indeterminate ancestry)
gnomAD genomes POPMAX AN	Total number of alleles in the population with the maximum AF
gnomAD genomes flag	Information from gnomAD genome data indicating whether the variant falling within low-complexity (lcr) or segmental duplication (segdup) or decoy regions. The flag can be either. For high-quality PASS or not reported/polymorphic in gnomAD exomes, ‘lcr’ for within lcr, ‘segdup’ for within segdup, or ‘decoy’ for with decoy region
Integrated confidence value	0 - highly significant scores (approx. *p* < 0.003); 1 – significant scores (approx. *p* < 0.05); 2 – informative scores (approx.*p* < 0.25); 3 – other scores (approx. *p* > = 0.25)
Integrated fitCons score	fitCons score predicts the fraction of genomic positions belonging to a specific function class (defined by epigenomic ‘fingerprint’) that are under selective pressure. Scores range from 0 to 1, with a larger score indicating a higher proportion of nucleic sites of the functional class the genomic position belong to are under selective pressure, therefore more likely to be functional important. Integrated (i6) scores are integrated across three cell types (GM12878, H1-hESC and HUVEC)
Integrated fitCons rankscore	The rankscore is the ratio of the rank of the score over the total number of integrated fitCons scores in dbNSFP
phastCons100way vertebrate	phastCons conservation score based on the multiple alignments of 100 vertebrate genomes (including human). The larger the score, the more conserved the site. Scores range from 0 to 1.
phastCons100way vertebrate rankscore	The rankscore is the ratio of the rank of the score over the total number of phastCons100way_vertebrate scores in dbNSFP
phastCons17way primate	A conservation score based on 17way alignment primate set. The larger the score, the more conserved the site. Scores range from 0 to 1.
phastCons17way primate rankscore	The rank of the phastCons17way_primate score among all phastCons17way_primate scores in dbNSFP
phastCons30way mammalian	phastCons conservation score based on the multiple alignments of 30 mammalian genomes (including human). The larger the score, the more conserved the site. Scores range from 0 to 1
phastCons30way mammalian rankscore	The rankscore is the ratio of the rank of the score over the total number of phastCons30way_mammalian scores in dbNSFP
phyloP100way vertebrate	phyloP (phylogenetic p-values) conservation score based on the multiple alignments of 100 vertebrate genomes (including human). The larger the score, the more conserved the site
phyloP100way vertebrate rankscore	The rankscore is the ratio of the rank of the score over the total number of phyloP100way_vertebrate scores in dbNSFP
phyloP17way primate	A conservation score based on 17way alignment primate set, the higher the more conservative
phyloP17way primate rankscore	The rank of the phyloP17way_primate score among all phyloP17way_primate scores in dbNSFP
phyloP30way mammalian	phyloP (phylogenetic p-values) conservation score based on the multiple alignments of 30 mammalian genomes (including human). The larger the score, the more conserved the site
phyloP30way mammalian rankscore	The rankscore is the ratio of the rank of the score over the total number of phyloP30way_mammalian scores in dbNSFP
rs dbSNP	rs number from dbSNP 151
PVS1 contrib	Null variant in a gene where loss of function (LOF) is a known mechanism of disease.
PS1 contrib	Same amino acid change as a previously established pathogenic variant regardless of nucleotide change.
PS2 contrib merged	*De novo* (both maternity and paternity confirmed) in a patient with the disease and no family history.
PS3 contrib	Well-established *in vitro* or *in vivo* functional studies supportive of a damaging effect on the gene or gene product.
PS4 contrib	The prevalence of the variant in affected individuals is significantly increased compared to the prevalence in controls.
PM1 contrib	Located in a mutational hot spot and/or critical and well-established functional domain (e.g. active site of an enzyme) without benign variation.
PM2 contrib	Absent from controls (or at extremely low frequency if recessive) (see Appendix A1) in exome sequencing Project, 1000 genomes or ExAC.
PM4 contrib	Protein length changes due to in-frame deletions/insertions in a non-repeat region or stop-loss variants.
PM5 contrib	Novel missense change at an amino acid residue where a different missense change determined to be pathogenic has been seen before.
PP2 contrib	Missense variant in a gene that has a low rate of benign missense variation and where missense variants are a common mechanism of disease.
PP3 contrib	Multiple lines of computational evidence support a deleterious effect on the gene or gene product (conservation, evolutionary, splicing impact, etc).
PP5 contrib	Reputable source recently reports variant as pathogenic but the evidence is not available to the laboratory to perform an independent evaluation.
BS1 contrib	Allele frequency is greater than expected for disorder.
BS2 contrib	Observed in a healthy adult individual for a recessive (homozygous), dominant (heterozygous), or X-linked (hemizygous) disorder with full penetrance expected at an early age.
BS3 contrib	Well-established *in vitro* or *in vivo* functional studies shows no damaging effect on protein function or splicing.
BP1 contrib	Missense variant in a gene for which primarily truncating variants are known to cause disease.
BP3 contrib	In-frame deletions/insertions in a repetitive region without a known function.
BP4 contrib	Multiple lines of computational evidence suggest no impact on gene or gene product (conservation, evolutionary, splicing impact, etc).
BP6 contrib	Reputable source recently reports variant as benign but the evidence is not available to the laboratory to perform an independent evaluation.
BP7 contrib	A synonymous (silent) variant for which splicing prediction algorithms predict no impact to the splice consensus sequence nor the creation of a new splice site AND the nucleotide is not highly conserved.
BA 1 contrib	Allele frequency is above 5 % in exome sequencing Project, 1000 genomes, or ExAC.
ALFA Total AC	Allele count for each ALT allele for the total (global) across all populations.
ALFA Total AF	The ratio of the allele count for each ALT allele for the population over the total allele count for the population, including REF
CCR Percentile	Percentile ranges from 90 to 100. 100 represents complete constraint, the highest constrained region in the model.
CCR Synonimous variant Density	A calculation of the synonymous variant density of the CCR region. Used variants that were SNPs and did not change amino acids or stop/start codons. Allowed multiple alleles at same bp.
CCR CpG	CpG dinucleotide density of the whole CCR region.
CCR cov score	The score of length scaled by coverage proportion at 10× for each base pair.
CCR residual	Raw residual value from the linear regression model.
CCR residual Percentile	Raw residual percentile, not weighted by proportion of exome represented.
CHASMplus P-value	P-value reflects the statistical significance of obtaining the acheived or higher CHASMplus score.
CHASMplus score	High scores reflect a greater likelihood that a mutation is a driver (scores range from 0.0 to 1.0).
CHASMplus transcript	Transcript ID.
CIViC description	Clinical interpretations of Vartiants in cancer description.
CIViC clinical Actionability score	Represents the accumulation of evidence.
CIViC diseases	Diseases names.
CIViC ID	ID number to link in CIViC database.
CIViC gene description	Clinical interpretations of Vartiants in cancer gene description
CScape coding score	Scores are p-values. Scores above 0.5 are predicted to be deleterious, while those below 0.5 are predicted to be neutral or benign. P-values close to the extremes (0 or 1) are the highest-confidence predictions that yield the highest accuracy.
CGC class	Driver class.
CGC inheritance	Somatic or germline inheritance.
CGC tumor type (Somatic)	Type of tumor according to location (caused by somatic mutations).
CGC tumor type (germline)	Type of tumor according to location (caused by germline mutations).
CGC link	Link to GCG database.
Cancer gene Landscape class	Class. Oncogenes and tumor supressor genes described by Vogelstein et al.
CVDKP IBS	Irritable bowel syndrome.
CVDKP CAD	Coronary artery disease.
CVDKP BMI	Body mass index and obesity.
CVDKP Atrial Fibrillation	Atrial Fibrillation.
CVDKP diabetes	Type 2 Diabetes.
DGI category	Gene categories in DGIdb refers to a set of genes belonging to a group that is deemed to be potentially druggable.
DGI interaction	An interaction type describes the nature of the association between a particular gene and drug.
DGI name	Drug name.
DGI score	A higher score indicates that there is a greater interaction between the drug and the gene. The score depends on numbers of drug and gene partners, as well as number of supporting publications and sources.
DGI ChEMBL	ChEMBL ID. ChEMBL is a manually curated database of bioactive molecules with drug-like properties.
DGI PMID	Link to literature.
Ensembl regulatory build region	Regions that are predicted to regulate gene expression.
Ensembl regulatory build ensr	An ensembl stable ID consists of five parts; ENS(species)(object type)(identifier).(version).
ESS gene	Essential or non-essential genes.
ESS gene indispensability score	A probability prediction of the gene being essential.
ESS gene indispensability prediction	Essential (E) or loss-of-function tolerant (N) based on Gene_indispensability_score.
GRASP NHLBI	NHLBI key.
GRASP PMID	PubMed ID.
GRASP phenotype	Phenotype.
GWAS catalog disease	Disease/Trait.
GWAS catalog Odds Ratio/Beta CObv/Expff	Odds Ratio/Beta Coeff.
GWAS catalog P-value	P-value.
GWAS catalog PMID	PubMed ID.
GWAS catalog initial sample	Initial sample.
GWAS catalog replication sample	Replication sample.
GWAS catalog risk allele	Risk allele.
GWAS catalog confidence interval	Confidence interval.
GO biological process name	GO biological process name.
GO biological process ID	GO biological process ID.
GO Cellular component name	GO Cellular component name.
GO Cellular component ID	GO Cellular component ID.
GO molecular function name	GO molecular function name.
GO molecular function ID	GO molecular function ID.
LitVar rs ID	Reference SNP ID.
LoFtool score	A percentile score for gene intolerance to functional change. The lower the score the higher gene intolerance to functional change.
NCBIGene description	NCBIGene description.
NCBIGene entrez	NCBIGene entrez.
RVIS EVS	Residual variation intolerance score, a measure of intolerance of mutational burden, the higher the score the more tolerant to mutational burden the gene is. Based on EVS (ESP6500) data.
RVIS Percentile EVS	The percentile rank of the gene based on RVIS, the higher the percentile the more tolerant to mutational burden the gene is. Based on EVS (ESP6500) data.
RVIS FDR ExAC	A gene’s FDR p-value for preferential LoF depletion among ExAC.
RVIS ExAC	ExAC-based RVIS; setting ‘common’ MAF filter at 0.05 % in at least one of the six individual ethnic strata from ExAC.
RVIS Percentile ExAC	Genome-Wide percentile for the new ExAC-based RVIS; setting ‘common’ MAF filter at 0.05 % in at least one of the six individual ethnic strata from ExAC.
SpliceAI Acceptor gain score	Probability of the variant being splice-altering. Cutoffs for binary prediction are, 0.2 (high recall), 0.5 (recommended), and 0.8 (high precision).
SpliceAI Acceptor loss score	Probability of the variant being splice-altering. Cutoffs for binary prediction are, 0.2 (high recall), 0.5 (recommended), and 0.8 (high precision).
SpliceAI Donor gain score	Probability of the variant being splice-altering. Cutoffs for binary prediction are, 0.2 (high recall), 0.5 (recommended), and 0.8 (high precision).
SpliceAI Donor loss score	Probability of the variant being splice-altering. Cutoffs for binary prediction are, 0.2 (high recall), 0.5 (recommended), and 0.8 (high precision).
SpliceAI Acceptor gain position	Location where splicing changes relative to the variant position (positive values are downstream of the variant, negative values are upstream).
SpliceAI Acceptor loss position	Location where splicing changes relative to the variant position (positive values are downstream of the variant, negative values are upstream).
SpliceAI Donor gain position	Location where splicing changes relative to the variant position (positive values are downstream of the variant, negative values are upstream).
SpliceAI Donor loss position	Location where splicing changes relative to the variant position (positive values are downstream of the variant, negative values are upstream).
VCF INFO Phred	VCF info Phred.
VCF INFO filter	VCF info filter.
VCF INFO zygosity	VCF info Zygosity: hom (homozygous) or het (heterozygous).
VCF INFO Alternate reads	Count of alternate reads.
VCF INFO Total reads	Count of total reads.
VCF INFO AF	VCF info AF.
dbscSNV AdaBoost	AdaBoost score. If the score >0.6 , it predicts that the splicing will be changed, otherwise it predicts the splicing will not be changed.
dbscSNV random forest	Random forest score. If the score >0.6 , it predicts that the splicing will be changed, otherwise it predicts the splicing will not be changed.
gnomAD gene transcript	Gene transcript ID.
gnomAD gene Obv/Exp LoF	Observed/Expected for loss of function variants.
gnomAD gene Obv/Exp Mis	Observed/Expected for missense variants.
gnomAD gene Obv/Exp Syn	Observed/Expected for synonymous variants.
gnomAD gene LoF Z-score	Z-score for loss of function variants.
gnomAD gene Mis Z-score	Z-score for missense variants.
gnomAD gene Syn Z-score	Z-score for synonymous variants.
gnomAD gene pLI	The probability of being loss-of-function intolerant (intolerant of both heterozygous and homozygous lof variants).
gnomAD gene pRec	The probability of being intolerant of homozygous, but not heterozygous lof variants.
gnomAD gene pNull	The probability of being tolerant of both heterozygous and homozygous lof variants.
p(HI)	Estimated probability of haploinsufficiency of the gene.
